# Identification of factors associated with stillbirth in Zimbabwe – a cross sectional study

**DOI:** 10.1186/s12884-021-04102-y

**Published:** 2021-09-29

**Authors:** Kushupika Dube, Tina Lavender, Kieran Blaikie, Christopher J. Sutton, Alexander E. P. Heazell, Rebecca M. D. Smyth

**Affiliations:** 1 Mpilo School of Midwifery, PO Box 2096, Vera Road, Bulawayo, Zimbabwe; 2 Centre for Childbirth, Women’s and Newborn Health, Liverpool School of Tropical Medicine, Liverpool, UK; 3 Centre for Biostatistics, School of Health Sciences, University of Manchester, Manchester, UK; 4 Maternal and Fetal Health Research Centre, School of Medical Sciences, Faculty of Biological, Medical and Human Sciences, University of Manchester, Manchester, UK; 5 National University of Science & Technology, P. O. Box AC 939, Ascot, Bulawayo, Zimbabwe; 6 Division of Nursing, Midwifery and Social Work, School of Health Sciences, The University of Manchester, Manchester, UK

## Abstract

**Introduction:**

98% of the 2.6 million stillbirths per annum occur in low and middle income countries. However, understanding of risk factors for stillbirth in these settings is incomplete, hampering efforts to develop effective strategies to prevent deaths.

**Methods:**

A cross-sectional study of eligible women on the postnatal ward at Mpilo Hospital, Zimbabwe was undertaken between 01/08/2018 and 31/03/2019 (*n* = 1779). Data were collected from birth records for maternal characteristics, obstetric and past medical history, antenatal care and pregnancy outcome. A directed acyclic graph was constructed with multivariable logistic regression performed to fit the corresponding model specification to data comprising singleton pregnancies, excluding neonatal deaths (*n* = 1734), using multiple imputation for missing data. Where possible, findings were validated against all women with births recorded in the hospital birth register (*n* = 1847).

**Results:**

Risk factors for stillbirth included: previous stillbirth (29/1691 (2%) of livebirths and 39/43 (91%) of stillbirths, adjusted Odds Ratio (aOR) 2628.9, 95% CI 342.8 to 20,163.0), antenatal care (aOR 44.49 no antenatal care vs. > 4 antenatal care visits, 95% CI 6.80 to 291.19), maternal medical complications (aOR 7.33, 95% CI 1.99 to 26.92) and season of birth (Cold season vs. Mild aOR 14.29, 95% CI 3.09 to 66.08; Hot season vs. Mild aOR 3.39, 95% CI 0.86 to 13.27). Women who had recurrent stillbirth had a lower educational and health status (18.2% had no education vs. 10.0%) and were less likely to receive antenatal care (20.5% had no antenatal care vs. 6.6%) than women without recurrent stillbirth.

**Conclusion:**

The increased risk in women who have a history of stillbirth is a novel finding in Low and Middle Income Countries (LMICs) and is in agreement with findings from High Income Countries (HICs), although the estimated effect size is much greater (OR in HICs ~ 5). Developing antenatal care for this group of women offers an important opportunity for stillbirth prevention.

**Supplementary Information:**

The online version contains supplementary material available at 10.1186/s12884-021-04102-y.

## Introduction

Despite advances in maternal and child health since the Millennium Development Goals, stillbirth remains a significant global health problem, with an estimated 2.6 million stillbirths per year, of which 98% occur in low and middle-income countries (LMICs) [1]. The Every Newborn Action Plan (ENAP) has set a target that the stillbirth rate in all countries should be lower than 12 per 1000 live births by 2030 [2]. To achieve this target the annual rate of reduction needs to accelerate from 1.4 to 4% [3]. Prevention of stillbirth requires information about risk factors in order that intervention strategies can be prioritised, developed and tested.

Systematic reviews of risk factors in LMICs have found a paucity of studies to inform estimates. One review including 142 studies from LMICS, found only 3 studies were conducted in low-income settings which have the greatest burden of stillbirth [4]. Comparison between studies is hampered by variation in the definition of stillbirth (or that definitions were not reported). Nevertheless, factors associated with increased risk of stillbirth include: maternal age (≥35 years or < 20 years), nulliparity or grand mutliparity, presence of maternal medical conditions, history of stillbirth, access to antenatal and intrapartum care, mode of birth and social factors such as rural location, low socioeconomic and educational status [4]; Importantly, this systematic review was not able to assess whether these risks were independent of one another. Furthermore, reported effect sizes vary between populations, as does the stillbirth rate. The low number of studies from low-income settings and variation in effect size between populations indicate that further studies are required to ensure risk factors are better understood before embarking on preventative strategies. It is anticipated that this will facilitate investment in interventions which are likely to yield the greatest benefit, which is important in settings where resource constraints preclude widespread adoption of interventions known to reduce stillbirths and neonatal deaths.

Zimbabwe is currently one of the world’s poorest countries with a debt >$12 billion and GDP of $31 billion in 2018 [5]. In 2015, the stillbirth rate in Zimbabwe was 21 per 1000 births [6]. Zimbabwe has both public and private healthcare systems, with the majority of basic care provided in rural district clinics and specialized care provided in urban settings. To achieve the target set out by ENAP Zimbabwe needs to attain an annual rate of reduction of 3.8% from 2015 to 2030. To obtain data about factors associated with stillbirth in Zimbabwe we conducted an observational study of women giving birth at Mpilo Hospital, Bulawayo, a tertiary-level government hospital with approximately 10,000 births per year.

## Methods

A retrospective cross-sectional study of women giving birth at Mpilo hospital was undertaken between 1st August 2018 and 31st March 2019. The definition of stillbirth employed in data collection was the WHO definition of a baby born with no signs of life with a gestation ≥28 weeks or a birthweight ≥1000 g when gestation was unknown; gestation for stillbirth was recorded as the gestation at presentation/diagnosis of stillbirth. An a priori sample size calculation determined that with a frequency of stillbirth of 3% (the rate reported at Mpilo hospital in 2017–2018) 1802 participants would be required to allow exploration of six explanatory variables in multivariable regression. Mpilo Hospital has approximately 10,000 births per year, thus it was initially anticipated that a 3-month sample of all births would achieve the requisite sample size. Prior to commencing the study ethical approval was obtained from The University of Manchester Research Ethics Committee (UREC 2018–4229-6699) and the Medical Research Council of Zimbabwe (MRC/E/203).

Anonymised data were collected about maternal demographic characteristics, past obstetric and medical history, antenatal care and development of complications in the index pregnancy and pregnancy outcome. Severe maternal pregnancy complications included any of: severe pre-eclampsia / eclampsia, sepsis, uterine rupture / obstructed labour, antepartum haemorrhage and anaemia. The case report form was largely informed by the WHO Making Every Baby Count Stillbirth and Neonatal Death Case Review form [7]; the definitions for each complication were those applied in local protocols derived from WHO standards. Data were recorded by two research assistants and entered into a specifically designed database in Research Electronic Data Capture (REDCap) hosted at The University of Manchester (https://www.project-redcap.org/). REDCap is a secure, web-based application designed to support data capture for research studies.

### Protocol deviations

We originally planned to obtain information on all births occurring during the study period by collecting data from women’s records in the immediate period after birth (http://lamrn.org/publications-resources/). This required data to be obtained from multiple sources; including the birth register and case notes which were held at either the postnatal ward or hospital records office. As births occurred continuously and research assistants were present 5 days a week therefore it was not possible to collect data synchronously, therefore information was sought several days after birth when records were no longer accessible. This resulted in incomplete case ascertainment and extension of the planned data collection period. The sample of eligible women obtained from the postnatal ward during the study period (Sept 2018 – March 2019) was obtained was *n* = 1779.

As this sample did not include all births, where possible findings were compared with those of all eligible women with births recorded in the hospital birth register between 1st August and 30th September 2018 (*n* = 1847). The birth register dataset recorded information on each birth (antenatal care, parity, HIV status, gestation, mode of birth, birthweight, infant sex, live/stillbirth and season of birth). Data from the birth register were transferred into REDCap.

### Statistical analysis

**Fig. 1:**
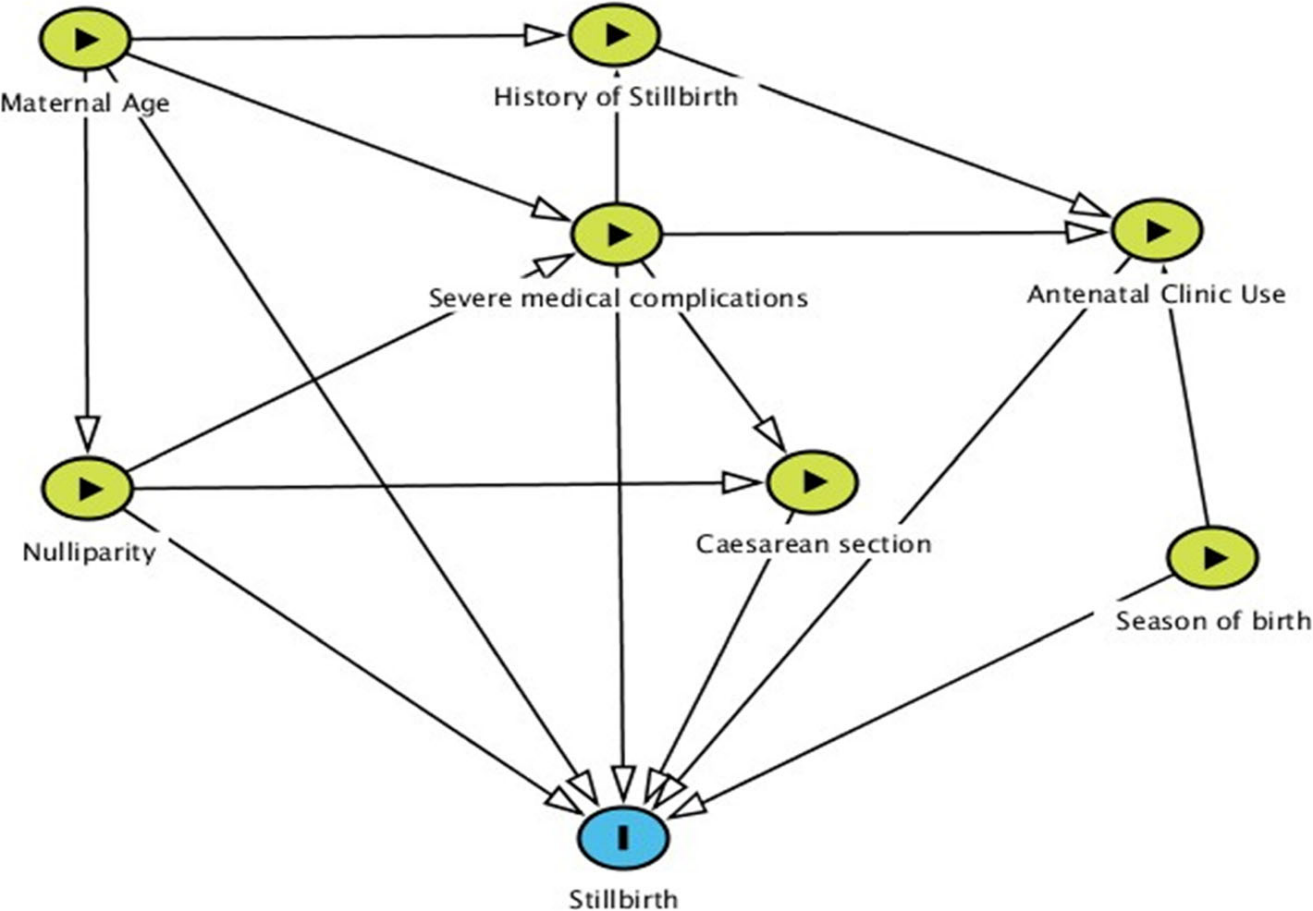
Directed acyclic graph to identify potential relationships between social, environmental and medical factors and stillbirth. Factors in blue are outcome variables, those in green with an arrow are exposures of interest. Diagram created in Dagitty Version 3.0^8^

A random sample of records in the postnatal ward cohort (10%) were double entered and discrepancies reviewed. The discrepancy rate was 0.51%; these were corrected and no further action was required. Statistical analysis was undertaken in R Version 3.5.1 [8]. Descriptive statistics were produced outlining the overall characteristics of each sample, as well as the characteristics of women whose pregnancy resulted in a livebirth or stillbirth separately. Crude Odds Ratio (OR) estimates were produced to explore the relationship between variables of interest and birth outcome (livebirth or stillbirth), with these estimates then compared descriptively between samples. Subsequently, a multivariate logistic regression model was developed for stillbirth, this allowed for adjustment of potential confounding factors, with the specific variables (maternal age, nulliparity, history of stillbirth, level of antenatal care (ANC) use, season of birth, birth by Caesarean section, and severe maternal complications) included in this model based on clinical knowledge informing a directed acyclic graph (DAG) (Fig. 1) [9]. The DAG was initially made including all factors considered in our theoretical model (Supplementary Figure 1), but in order to improve model stability and cut down the number of include parameters, variables were removed guided by subject matter expertise (keeping the variables a priori thought to be most important), availability of information (e.g. there was no reliable measure of socioeconomic status) as well as a decision to focus on antecedents of stillbirth. To account for missing data, multiple imputation by chained equations (MICE) (using the variables described above with the addition of medical referral, gravidity, HIV status, presence of reduced fetal movements, use of an ambulance, anaemia, marital status, gestational age at birth, smoking status, level of education, employment, partograph completion and urban or rural location) was used in our primary analysis [10]. This method enabled us to retain all participants in our analysis maintaining statistical power by avoiding the exclusion of cases with information on some, but not all variables. It replaces missing observation data with values we may expect based on available participant characteristics, assuming the information missing is ‘Missing At Random’. [11] As a sensitivity analysis, a model was produced restricting our sample to complete cases.

### Patient and public involvement

The study was supported by community engagement and involvement (CEI) and stakeholder groups prior to data collection procedures in the form of focus group discussions. Stakeholders were local academics, clinicians, managers and ministry of health representatives, recruited via established networks and CEIs compromised bereaved parents. Outcome measures were developed and informed by their priorities. CEI members raised community awareness of the study and involved in the process of dissemination via mediums that are accessible to all community members.

## Results

**Fig. 2:**
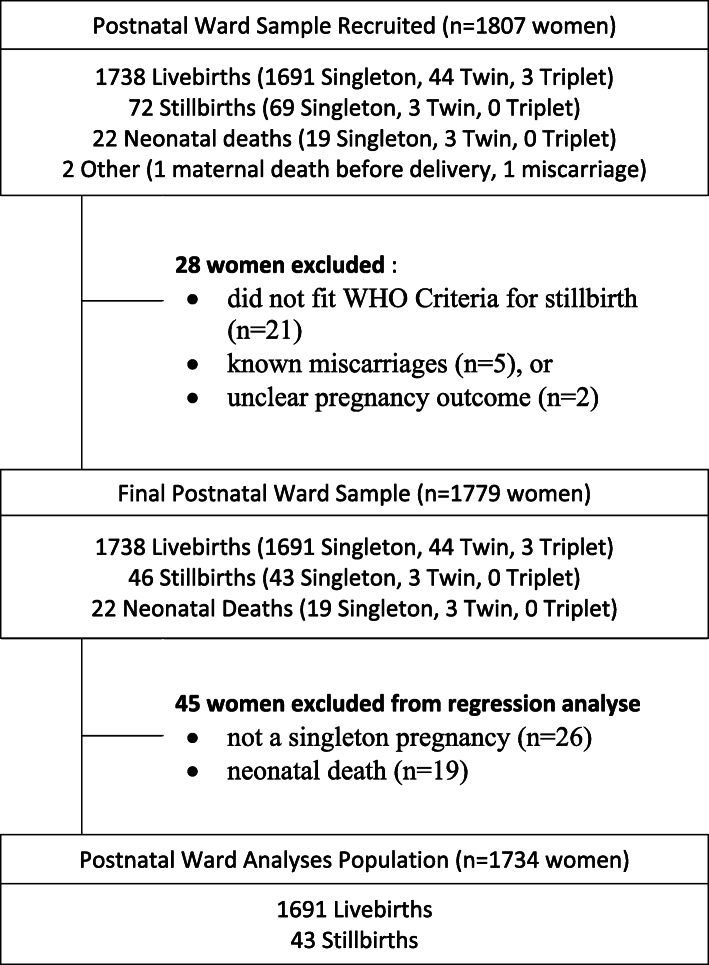
Study flowchart for the sample recruited from postnatal ward reporting reasons for exclusion from the primary study analysis

During the study period there were 6870 births, from which data were collected from 1807 participants on the postnatal ward (26.3% of all births, Table 1). This sample included 72 women who were initially recorded as having a stillbirth. Subsequently, 26 of these cases were excluded as they did not meet the WHO criteria for stillbirth and 2 further cases were excluded as the outcome was uncertain or the gestation at loss was unknown. A further 45 births were excluded from the regression analysis because they were from multiple pregnancies (*n* = 26) or the baby died in the neonatal period (*n* = 19). The final postnatal ward analysis included 1734 women, 1691 of whom had a live birth and 43 who had a stillbirth (Fig. 2). This gives a stillbirth rate of 2.5% which was slightly less than that in the whole maternity population of 3.2% (219/6870 births).

**Table 1:** Singleton pregnancy model findings based on data from postnatal ward sample (percentages exclude unknown cases)

	Livebirth	Stillbirth	Unadjusted CC OR (95% CI)	***p***-value	Adjusted MI OR (95% CI)	***p***-value
	***N*** = 1691	***N*** = 43				
Mother’s age in years						
Mean (SD)	26.8 (6.6)	28.0 (7.1)	1.03 (0.98–1.07)	0.23	1.04 (0.95–1.14)	0.41
Unknown	0	0				
Nulliparous						
No	1090 (64%)	41 (95%)	1		1	
Yes	601 (36%)	2 (5%)	0.09 (0.01–0.29)	< 0.001	3.51 (0.37–33.30)	0.27
Unknown	0	0				
History of stillbirth						
No	1662 (98%)	4 (9%)	1		1	
Yes	29 (2%)	39 (91%)	558.8 (208.7–1955.8)	< 0.001	2628.9 (342.8–20,163.0)	< 0.001
Unknown	0	0				
Number of antenatal care visits						
> 4	533 (35%)	6 (18%)	1		1	
1–4	861 (57%)	18 (53%)	1.86 (0.77–5.15)	0.19	5.35 (1.31–21.87)	0.02
None	110 (7%)	10 (29%)	8.08 (2.94–24.17)	< 0.001	44.49 (6.80–291.19)	< 0.001
Unknown	187	9				
Season of birth						
Mild	1081 (64%)	18 (42%)	1		1	
Cold	239 (14%)	13 (30%)	3.27 (1.55–6.72)	0.001	14.29 (3.09–66.08)	0.001
Hot	371 (22%)	12 (28%)	1.94 (0.90–4.03)	0.08	3.39 (0.86–13.27)	0.08
C-section delivery						
No	1211 (72%)	28 (65%)	1		1	
Yes	480 (28%)	15 (35%)	1.35 (0.70–2.52)	0.35	0.65 (0.21–2.06)	0.47
Severe maternal complications						
No	1423 (86%)	19 (54%)	1		1	
Yes	223 (14%)	16 (46%)	5.37 (2.69–10.60)	< 0.001	7.33 (1.99–26.92)	0.003
Unknown	45	8				
Marital status						
Not married	330 (21%)	6 (15%)	1		-	
Married	1213 (79%)	34 (85%)	1.54 (0.69–4.11)	0.33		
Unknown	148	3				
Level of education						
None or primary	135 (10%)	4 (17%)	1		-	
Secondary	1132 (84%)	17 (74%)	0.51 (0.18–1.78)	0.23		
Higher or voc.	81 (6%)	2 (9%)	0.83 (0.11–4.37)	0.84		
Unknown	343	20				
Formal employment						
No	1199 (89%)	22 (92%)	1		-	
Yes	146 (11%)	2 (8%)	0.75 (0.12–2.57)	0.69		
Unknown	346	19				
Religion						
Christian	1221 (96%)	30 (97%)	1		-	
Muslim or other	57 (4%)	1 (3%)	0.71 (0.04–3.43)	0.74		
Unknown	413	12				
Alcohol Consumption						
No	1497 (99%)	32 (100%)	-		-	
Yes	15 (1%)	0 (0%)				
Unknown	179	11				
Smoking status						
Non-smoker	1511 (99%)	32 (100%)	-		-	
Smoker	3 (< 1%)	0 (0%)				
Unknown	177	11				
HIV status						
Negative	1395 (83%)	33 (80%)	1		-	
Positive	286 (7%)	8 (20%)	1.18 (0.50–2.46)	0.68		
Unknown	10	2				
Anaemia						
No	1521 (98%)	32 (91%)	1		-	
Yes	35 (2%)	3 (9%)	4.07 (0.95–12.10)	0.03		
Unknown	135	8				
Syphilis during pregnancy						
No	1523 (96%)	31 (89%)	1		-	
Yes	58 (4%)	4 (11%)	3.39 (0.98–8.92)	0.03		
Unknown	110	8				
BMI during pregnancy						
Mean (SD)	28.4 (5.0)	27.4 (5.4)	0.96 (0.87–1.05)	0.37	–	
Unknown	434	22				
Care available at nearest health facility						
Basic EmOC	551 (34%)	13 (34%)	1		-	
Home delivery or first-aid	5 (< 1%)	0	-			
Comp. EmOC	1067 (66%)	25 (66%)	0.99 (0.51–2.02)	0.98		
Unknown	68	5				
Distance from home to referral hospital (minutes)						
≤ 30	978 (97%)	23 (100%)	1		-	
31–60	19 (2%)	0 (0%)	-			
61–120	9 (1%)	0 (0%)	-			
120+	2 (< 1%)	0 (0%)	-			
Unknown	683	20				
Doctor or obstetrician present at delivery						
No	1205 (71%)	26 (60%)	1		-	
Yes	486 (29%)	17 (40%)	1.62 (0.86–2.99)	0.13		

*CC* Complete case, *MI* Multiple Imputation, *OR* Odds Ratio, *CI* Confidence Interval, *SD* Standard Deviation

The majority of women who had a singleton stillbirth had no fetal heart at the time of admission (34/38, 89.5% (unknown in 5 cases)) and (35/43) 81% of stillborn babies had skin changes from in utero retention indicating that was largely a population of antepartum stillbirths. Although the majority of women who had a stillbirth had comprehensive emergency obstetric care (EmOC) (25/38, 65.8%, (unknown in 5 cases)), only 6/34 (17.6%) had attended more than 4 antenatal appointments. Eight cases (19.5% (unknown in 2 cases)) were HIV positive and 10 (23.3%) were recorded as having preeclampsia or eclampsia. The cause of stillbirth was recorded as unexplained in 34/43 (79.1%) cases, 3 (7.0%) were recorded as resulting from antepartum haemorrhage, 2 (4.7%) from preeclampsia/eclampsia and 2 (4.7%) cases each of uterine rupture and cord prolapse.

Analysis of the postnatal ward cohort found that history of stillbirth (adjusted odds ratio [aOR] 2628.9, 95% CI 342.8 to 20,163.0) and inadequate antenatal care compared to > 4 ANC appointments (No ANC aOR 44.49, 95% CI 6.80 to 291.19, 1–4 ANC appointments aOR 5.35, 95% CI 1.31 to 21.87) were independently significantly associated with increased risk of stillbirth (Table 2). The presence of severe maternal complications (aOR 7.33, 95% CI 1.99 to 26.92) and giving birth in the cold season compared to the mild season (aOR 14.29, 95% CI 3.09 to 66.08) were also significantly more frequent in women who had a stillbirth. There was no significant difference (*p* = 0.27, chi-square test) between the percentages of women with severe maternal complications in the cold, mild or hot seasons. However, as would be expected given that the corresponding aORs are greater than the cORs in Table 1, the observed percentage of women suffering prenatal complications in the cold season (27/247, 10.9%) was lower than in the mild (156/908, 14.7%) or hot (56/370, 15.1%).

**Table 2:** Comparison of singleton pregnancy characteristics between groups identified from the birth register and postnatal ward sample (percentages exclude unknown cases)

	Birth Register				Post-natal Ward			
	Livebirth	Stillbirth	CC OR (95% CI)	***p***-value	Livebirth	Stillbirth	CC OR (95% CI)	***p***-value
	***N*** = 1746	***N*** = 39			***N*** = 1691	***N*** = 43		
Mother’s age in years								
Mean (SD)	26.0 (6.5)	27.6 (6.8)	1.04 (0.99–1.08)	0.16	26.8 (6.6)	28.0 (7.1)	1.03 (0.98–1.07)	0.23
Unknown	16	2			0	0		
Nulliparous								
No	1093 (63%)	27 (73%)	1		1090 (64%)	41 (95%)	1	
Yes	643 (37%)	10 (27%)	0.63 (0.29–1.27)	0.22	601 (36%)	2 (5%)	0.09 (0.01–0.29)	< 0.001
Unknown	10	2			0	0		
Gravida								
1–2	1099 (63%)	18 (49%)	1		1036 (61%)	22 (51%)	1	
3–4	523 (30%)	15 (41%)	1.75 (0.86–3.50)	0.11	527 (31%)	17 (40%)	1.52 (0.79–2.88)	0.20
5 +	113 (7%)	4 (11%)	2.16 (0.62–5.91)	0.17	128 (8%)	4 (9%)	1.47 (0.43–3.92)	0.48
Unknown	11	2			0	0		
Any antenatal care								
No	176 (10%)	6 (17%)	1		110 (7%)	10 (23%)	1	
Yes	1536 (90%)	30 (83%)	0.57 (0.25–1.54)	0.22	1581 (93%)	33 (77%)	0.23 (0.11–0.50)	< 0.001
Unknown	34	3			0	0		
HIV status								
Negative	1403 (83%)	31 (91%)	1		1395 (83%)	33 (80%)	1	
Positive	296 (17%)	3 (9%)	0.46 (0.11–1.30)	0.20	286 (17%)	8 (20%)	1.18 (0.50–2.46)	0.68
Unknown	47	5			10	2		
Tetanus vaccination								
No	76 (4%)	7 (19%)	1		48 (3%)	5 (15%)	1	
Yes	1635 (96%)	29 (81%)	0.19 (0.09–0.49)	< 0.001	1506 (97%)	28 (85%)	0.18 (0.07–0.54)	< 0.001
Unknown	35	3			137	10		
History of stillbirth								
No	1722 (99%)	34 (87%)	1		1662 (98%)	4 (9%)	1	
Yes	14 (1%)	5 (13%)	18.09 (5.60–50.34)	< 0.001	29 (2%)	39 (91%)	558.8 (208.7–1955.8)	< 0.001
Unknown	10	0			0	0		
Caesarean-section delivery								
No	1195 (68%)	28 (72%)	1		1211 (72%)	28 (65%)	1	
Yes	551 (32%)	11 (28%)	0.85 (0.40–1.68)	0.66	480 (28%)	15 (35%)	1.35 (0.70–2.52)	0.35
Gestational age at birth in weeks								
Mean (SD)	38.6 (2.1)	35.1 (3.8)	0.71 (0.64–0.79)	< 0.001	38.6 (2.2)	35.4 (3.6)	0.70 (0.64–0.78)	< 0.001
Unknown	324	11			168	8		

*CC* Complete case, *OR* Odds Ratio, *CI* Confidence Interval, *SD* Standard Deviation

When the sensitivity analysis was undertaken in complete cases only (*n* = 1464 live births and *n* = 28 stillbirths) the distribution of cases and controls appeared to be similar (Supplementary Table 1), though the small numbers of cases reduced the reliability and precision of estimates in the multivariable model, hence has not been presented. When cases with missing data were excluded, the conclusions remained the same, with history of stillbirth, lack of antenatal care and the presence of severe maternal complications being significantly associated with stillbirth.

As we were not able to collect data on all births occurring in the time-period of the postnatal cohort study, our findings were validated on information available on a consecutive sample from the birth register (*n* = 1785, which included 39 stillbirths). The birth register had a similar proportion of singleton births (99% in both), stillbirths (2% vs. 3%) and neonatal deaths (2% vs 1%) to the postnatal cohort. In comparison to the population in the postnatal cohort, women whose outcomes were recorded from the birth register demonstrated similar effects (albeit smaller in magnitude) in univariate comparisons. Tetanus vaccination (crude OR (cOR) 0.19, 95% CI 0.09 to 0.49) and increasing gestational age (in week increments cOR 0.71, 95% CI 0.64 to 0.79)) were each associated with a significantly reduced risk of stillbirth and a history of stillbirth in a previous pregnancy was associated with significantly increased risk (cOR 18.09, 95%CI 5.60 to 50.34). However, maternal age, nulliparity and antenatal care were not significantly associated with stillbirth in the birth register dataset.

Due to the increased risk of stillbirth in women with a history of stillbirth in both datasets, we examined the cases of *recurrent* stillbirth (*n* = 39/43) from the postnatal ward cohort in more detail. Compared to women who had not had recurrent stillbirth (i.e. live births + non-recurrent stillbirth *n* = 1695) these mothers had similar ages, body mass index, gravidity and parity (Table 3). However, women who had recurrent stillbirths had higher gravidity (median 3 in women with recurrent stillbirth and 2 in non-recurrent stillbirth) and they gave birth earlier 35.7 weeks vs. 38.6 weeks, had a lower educational status and lived further from the nearest health facility. Syphilis was more common in those with recurrent stillbirth than those without (12.1% vs. 3.7%) as was anaemia (9.4% vs. 2.2%). Those with recurrent stillbirth are less likely to have antenatal care (79.5% vs. 93.4%), have fewer visits if they do have ANC, and do not tend to present in the first trimester of pregnancy. Similarly, tetanus vaccination was less common in those with recurrent stillbirth (86.7% vs. 96.9%) possibly due to less ANC use. Severe medical complications were more common in women with recurrent stillbirth (59.4% vs. 14.6%). Unfortunately, few cases of recurrent stillbirth had a recorded cause of death, but placental abnormalities were reported in 79.3% of women with recurrent stillbirth compared to 1.1% of live births.

**Table 3:** Maternal demographic and pregnancy characteristics in women who had recurrent stillbirths compared to those who did not have recurrent stillbirth (i.e. had two live births or a stillbirth followed by a live birth)

Singleton Pregnancy Population Characteristics		Recurrent Stillbirths	
		No (***n*** = 1695)	Yes (***n*** = 39)
Maternal Age (years)	Mean (SD)	26.8 (6.6)	27.7 (6.9)
BMI during pregnancy	Mean (SD)	28.4 (5.0)	27.6 (5.5)
	Unknown	437	19
Gestational Age at Birth (weeks)	Mean (SD)	38.6 (2.3)	35.7 (3.5)
	Unknown	170	6
Gravidity	Mean (SD)	2.4 (1.4)	2.9 (1.1)
	Median [IQR]	2.0 [1–3]	3.0 [2–4]
Parity	Mean (SD)	1.2 (1.3)	1.8 (1.1)
	Median [IQR]	2.0 [1–3]	1.0 [1–2]
Nulliparous	No	1092/1695 (64.4%)	39/39 (100.0%)
	Yes	603/1695 (35.6%)	0/39 (0.0%)
History of Stillbirth	No	1666/1695 (98.3%)	0/39 (0.0%)
	Yes	29/1695 (1.7%)	39/39 (100.0%)
Stillbirth	No	1691/1695 (99.8%)	0/39 (0.0%)
	Yes	4/1695 (0.2%)	39/39 (100.0%)
Education	None or Primary	135/1349 (10.0%)	4/22 (18.2%)
	Secondary	1133/1349 (84.0%)	16/22 (72.7%)
	Tertiary or Voc.	81/1349 (6.0%)	2/22 (9.1%)
	Unknown	346	17
Married	No	330/1546 (21.3%)	6/37 (16.2%)
	Yes	1216/1546 (78.7%)	31/37 (83.8%)
	Unknown	149	2
Formal Employment	No	1200/1346 (89.2%)	21/23 (91.3%)
	Yes	146/1346 (10.8%)	2/23 (8.7%)
	Unknown	349	16
HIV	No	1200/1346 (89.2%)	21/23 (91.3%)
	Yes	146/1346 (10.8%)	2/23 (8.7%)
	Unknown	349	16
Syphilis during pregnancy	No	1525/1583 (96.3%)	29/33 (87.9%)
	Yes	58/1583 (3.7%)	4/33 (12.1%)
	Unknown	112	6
Anaemia	No	1524/1559 (97.8%)	29/32 (90.6%)
	Yes	35/1559 (2.2%)	3/32 (9.4%)
	Unknown	136	7
Tetanus Vaccination	No	49/1557 (3.1%)	4/30 (13.3%)
	Yes	1508/1557 (96.9%)	26/30 (86.7%)
	Unknown	138	9
Any Antenatal Care	No	112/1695 (6.6%)	8/39 (20.5%)
	Yes	1583/1695 (93.4%)	31/39 (79.5%)
Number of ANC visits	> 4	533/1507 (35.4%)	6/31 (19.4%)
	1–4	862/1507 (57.2%)	17/31 (54.8%)
	None	112/1507 (7.4%)	8/31 (25.8%)
	Unknown	188	8
First Trimester antenatal care	No	1254/1278 (98.1%)	23/23 (100.0%)
	Yes	24/1278 (1.9%)	0/23 (0.0%)
	Unknown	417	16
Level of nearest care	Basic EmOC	552/1626 (34.0%)	12/35 (34.3%)
	Home Delivery. or First Aid	5/1626 (0.2%)	0/35 (0.0%)
	Comp. EmOC	1069/1626 (65.8%)	23/35 (65.7%)
	Unknown	69	4
Distance home to nearest health facility (minutes)	< 30	419/427 (98.1%)	6/7 (85.7%)
	31–60	3/427 (0.7%)	1/7 (14.3%)
	61–119	3/427 (0.7%)	0/7 (0.0%)
	120+	2/427 (0.5%)	0/7 (0.0%)
	Unknown	1268	32
Distance home to referral hospital (minutes)	< 30	981/1011 (97.0%)	20/20 (100.0%)
	31–60	19/1011 (1.9%)	0/20 (0.0%)
	61–119	9/1011 (0.9%)	0/20 (0.0%)
	120+	2/1011 (0.2%)	0/20 (0.0%)
	Unknown	684	19
Ambulance Use	No	1358/1595 (85.1%)	26/36 (72.2%)
	Yes	237/1595 (14.9%)	10/36 (27.8%)
	Unknown	100	3
Severe Maternal Complications	No	1425/1649 (86.4%)	17/32 (53.1%)
	Yes	224/1649 (13.6%)	15/32 (46.9%)
	Unknown	46	7
Placental Abnormalities	No	1412/1428 (98.9%)	6/29 (20.7%)
	Yes	16/1428 (1.1%)	23/29 (79.3%)
	Unknown	267	10
Fetal Heartbeat on Admission	No	23/1686 (1.4%)	31/35 (88.6%)
	Yes	1663/1686 (98.6%)	4/35 (11.4%)
	Unknown	9	4
Reduced Fetal Movement	No	1647/1689 (97.5%)	6/25 (24.0%)
	Yes	42/1689 (2.5%)	19/25 (76.0%)
	Unknown	6	14
Last Reported Fetal Movement	Before Admission	37/1687 (2.2%)	26/32 (81.2%)
	In Hospital	1650/1687 (97.8%)	6/32 (18.8%)
	Unknown	8	7
Mode of Delivery	SVD	1202/1695 (70.9%)	24/39 (61.5%)
	C-Section	481/1695 (28.4%)	14/39 (35.9%)
	Other	12/1695 (0.7%)	1/39 (2.6%)
Type of Stillbirth	Fresh	0/4 (0.0%)	8/38 (21.1%)
	Skin changes present	4/4 (100.0%)	30/38 (78.9%)
	Unknown	0	1
Reported Cause of Stillbirth	PE/Eclampsia/PIH	0/4 (0.0%)	2/39 (5.1%)
	Uterine Rupture	0/4 (0.0%)	2/39 (5.1%)
	Cord Prolapse	0/4 (0.0%)	2/39 (5.1%)
	APH	1/4 (25.0%)	2/39 (5.1%)
	Unexplained	3/4 (75.0%)	31/39 (79.5%)

## Discussion

This was a hypothesis-generating study to identify factors associated with stillbirth which could be amenable to modification to reduce stillbirth. We were able to determine factors associated with stillbirth in Zimbabwe in a sample of adequate size. Some of these factors were then validated in a sample of consecutive births taken from the birth register. This approach has enable us to identify some risk factors that have been reported previously in low-resource settings, such as lack of antenatal care, increasing maternal age, and presence of maternal medical complications. It has also identified risk factors which have not been widely reported in LMICs, including previous stillbirth and seasonality. This study has also demonstrated that the effect of some risk factors varied between different studies and locations (such as the effect of HIV infection).

### Strengths and limitations of this study

This study was strengthened by data collection from a large cohort of women by clinically trained staff who were familiar with local documentation and practice which enabled detailed information to be obtained. However, data acquisition could not keep pace with the number of births which could have introduced selection bias although notably the demographics characteristics of both cohorts were similar. Data collected were reliant upon information recorded in the antenatal and intrapartum case notes which was sometimes missing; the presence of data may be negatively influenced by disclosure of conditions which may be stigmatising (e.g. syphilis, previous stillbirth). The inclusion of local maternity staff in the study enabled nuanced discussions about the relationship between confounding factors which informed the DAG and the subsequent multivariable analysis.

The use of the DAG also enabled the researchers to identify limitations in our model. For example, our theoretical DAG identified socioeconomic status and cigarette smoking as potential confounding factors, but only limited data were available for these variables which meant they could not be accounted for in our model which may have introduced bias into our results.

Although the number of participants in the study was relatively large, the number of participants in some groups was small which may have limited the statistical power of this study to determine some risk factors i.e. would produce a type 2 statistical error. Therefore, it remains important to examine other potential risk factors for stillbirth in even larger datasets in settings, such as Zimbabwe, where there is a high burden of stillbirth.

### Regional context

The factors associated with stillbirth here are in agreement with those described by Aminu et al. in a systematic review of 142 studies of risk factors for stillbirth in LMICs, 49 of which were from Africa [4]. Due to variations employed in source studies and risk factors studied formal meta-analysis was not possible. In their narrative synthesis, the authors identified lack of antenatal care and previous stillbirth, which were amongst the largest independent effects in our study [4]. Notably, these effects are not unique to LMICs. Systematic reviews and meta-analysis of observational studies demonstrate a relationship between stillbirth and increasing maternal age, with the greatest effects seen in women ≥40 years of age [12]. A meta-analysis of studies from HICs found that women who have a history of stillbirth were more likely to have a stillbirth in a subsequent pregnancy [13]. The fact that these associations are present irrespective of setting suggests that they have their origins in human biology. However, their effect sizes vary which indicates that other local sociodemographic characteristics may moderate this increased risk. For example, the effect size of having a previous stillbirth in Zimbabwe was greater than reported in HICs. This may be because women with a previous stillbirth were more likely to live in a rural location and have less access to antenatal care.

Comparison with prior studies of stillbirth and perinatal death in Zimbabwe reveals variation in the stillbirth rate between different regions and time-periods ranging from 1.7 to 6.1% [14–17]. This could be attributable to various factors including changes in economic prosperity over time or differences in the urban/rural mix of population between sites. These studies report variation in factors associated with stillbirth. Crowther reviewed 53,665 births in Harare in 1983, of which 1204 were stillbirths, 17.0% of stillbirths were macerated of unknown cause, 14.0% were attributed to intrapartum asphyxia, 5.4% were associated with hypertensive disorders of pregnancy and 8.1% with antepartum haemorrhage [15]. Aiken reviewed 466 stillbirths at Mpilo Hospital from 1989 to 1990, describing causes of stillbirth were congenital syphilis (21.7%), birth asphyxia (23.8%), unexplained stillbirths (21.5%), congenital malformations (7.3%), pregnancy-induced hypertension (9.9%) and placental abruption (8.8%) [14]. A review of women with HIV infection conducted at the same time found 15% of women who had a stillbirth were HIV positive; HIV mothers had more stillbirths associated with syphilis and congenital infection [18]. Feresu et al. examined 985 stillbirths and 17,174 live births in Harare in 1997–1998 using a threshold of 20 weeks’ gestation and 500 g to define stillbirth; this study found maternal age ≥ 35 years, rural location and women who were unbooked for antenatal care were associated with stillbirth, in this population nulliparity was protective [16]. A population-based survey conducted in 2006–7 undertook verbal autopsy in 11 areas, one of which was near Bulawayo; 1296 stillbirths from 45,023 live births were reviewed. This report identified that maternal disease was thought to be causal in 7.8% of stillbirths [17]. Lastly, an interview study of 103 cases and 206 controls conducted in 2009 in Mashonaland found lack of education, labour complications, home birth, HIV infection and low birthweight (< 2.5 kg) were associated with stillbirth [19]. This region has a high proportion of people from the Apostolic church who have little engagement with medical services. These studies agree that poor access to maternity care, medical complications (particularly hypertension) and labour complications are important risk factors for stillbirth in Zimbabwe as they have been consistently observed over time.

Importantly, we observed a lower proportion of intrapartum stillbirth than reported previously which may have been due to a focus on reducing term stillbirth at Mpilo hospital in 2017 [20]; the levels reported in our study were consistent with these data from the preceding year in the maternity unit under study. We also did not observe an association between HIV-positive status and stillbirth reported in earlier studies [18, 19]. This may be because testing and antiretroviral therapy to reduce vertical transmission are embedded within contemporary maternity care in Zimbabwe.

A novel association with stillbirth in Zimbabwe described in this study was the association of stillbirth and birth in the cold or hot season compared to the mild season. A systematic review of 32 studies found that pregnancy length and birth outcomes were altered, particularly in summer and winter [21]. The four included studies that examined stillbirth found higher rates of stillbirth in winter (cold) from studies in the Northern Hemisphere and with summer (hot) in Australia [21]. One subsequent study examining the effects of seasonality in Nepal found the peak incidence of stillbirth was in January (cold). Thus, our findings appear consistent with other studies from the literature [22]. Further research is required to better understand whether this association is independent, or whether it is mediated by behavioural changes e.g. use of indoor stoves/fuel, difficulty accessing maternity care or alterations in diet.

### Clinical implications

The strong association between prior stillbirth and subsequent stillbirth in our study population was particularly striking. Of the women who had a stillbirth 39/43 (91%) had a previous stillbirth. This percentage was higher than a study from Malawi which found 62.7% of mothers who experienced a perinatal death had previously had a perinatal death [23]. Four other studies, three of which were conducted in Africa (Ghana, Nigeria and Zambia), found an association between stillbirth and a history of stillbirth. The crude effect sizes ranged from 1.94–5.7 [24–26]. The consistency of this observation suggests that it is robust and likely to occur in different LMICs. Recurrent stillbirth is particularly significant given the stigma and taboo that surround stillbirth in many societies; beliefs that stillbirth is the result of a curse or errant maternal behaviour are likely to be reinforced if a mother experiences recurrent deaths [27]. However, women who have a history of stillbirth may represent a group for whom care can be modified. After a stillbirth has occurred, women could be counselled about the importance of attending for antenatal care from an earlier time point in a subsequent pregnancy. Regular attendance in maternity services could ensure adequate screening for syphilis and hypertensive disorders of pregnancy, and potentially administration of prophylactic aspirin which reduces the risk of perinatal death [28, 29].

Our findings, and those of earlier studies, emphasise the importance of antenatal care as unbooked women have a significantly increased risk of stillbirth as well as increased risk of maternal and neonatal mortality. Since 2018, antenatal care in Zimbabwe has been free at the point of care, removing one barrier to accessing maternity care. However, additional services may require additional payment. Improvements in intrapartum care, including increased skilled birth attendants and access to Caesarean section, may have reduced the proportion of intrapartum stillbirths in our study population. Access to evidence-based interventions in antenatal and intrapartum care should continue to be prioritised, as their implementation will reduce stillbirths, neonatal and maternal deaths achieving a triple return on investment [27].

## Conclusion

This study demonstrates independent associations between a history of stillbirth, number of antenatal care visits, seasonality of birth, and the presence of maternal complications with stillbirth. As many of these risk factors have important effects on maternal and neonatal health as well, these factors deserve input from public health and maternity services. Provision of antenatal and intrapartum care is a priority, particularly in the case of women who have medical disorders or who have a history of stillbirth. The optimal means to identify women at highest risk to ensure they present to antenatal care with sufficient time to screen for and appropriately manage conditions needs to be ascertained, particularly because treatment in early pregnancy is most effective. Women who have had a stillbirth or a pregnancy complicated by severe medical problems (e.g. severe preeclampsia/eclampsia) should be sensitively advised to engage with antenatal care early in a subsequent pregnancy. Further studies are needed in LMICs to develop antenatal care strategies for women who have a history of stillbirth to minimise the risk of subsequent complications; a feasibility study exploring this strategy has been commenced at Mpilo hospital in 2019 (ISRCTN78733502) [30].

## Supplementary Information

**Additional file 1: Supplementary Figure 1.** Theoretical Directed Acyclic Graph to identify potential relationships between social, behavioural and medical factors and stillbirth. Factors in blue are outcome variables, those in green with an arrow are exposures of interest and those in grey are unmeasured factors. Diagram created in Dagitty Version Version 3.0.^8^ SES = Socioeconomic status, HIV – Human Immunodeficiency Virus, Syph/Tet – Syphilis serology and Tetanus vaccination.

**Additional file 2: Supplementary Table 1.** Singleton pregnancy model findings based on complete case data from postnatal ward sample (percentages exclude unknown cases).

## Data Availability

The datasets generated and/or analysed during the current study are not publicly available as ethical approval was not sought for their dissemination but are available from Professor Tina Lavender; Tina.Lavender@lstmed.ac.uk. Type of data: Anonymised quantitative. Available for 5 years following publication of results.

## Abbreviations

ANC: Antenatal care
aOR: adjusted Odds Ratio
CEI: Community Engagement and Involvement
cOR: crude Odds Ratio
DAG: Directed Acyclic Graph
EmOC: Emergency Obstetric Care
ENAP: Every Newborn Action Plan
GDP: Gross Domestic Product
HIC: High Income Countries
HIV: Human Immunodeficiency Virus
LMIC: Low and Middle Income Countries
MICE: Multiple imputation by chained equations
REDCap: Research Electronic Data Capture
WHO: World Health Organisation
