# Supplementary material for: Identification of factors associated with stillbirth in Zimbabwe – a cross sectional study
Source: BMC Pregnancy Childbirth. 2021 Sep 29;21:662. doi: 10.1186/s12884-021-04102-y (PMC8482658; doi:10.1186/s12884-021-04102-y)

**Supplementary Figure 1** Theoretical Directed Acyclic Graph to identify potential relationships between social, behavioural and medical factors and stillbirth. Factors in blue are outcome variables, those in green with an arrow are exposures of interest and those in grey are unmeasured factors. Diagram created in Dagitty Version Version 3.0.^8^ SES = Socioeconomic status, HIV – Human Immunodeficiency Virus, Syph/Tet – Syphilis serology and Tetanus vaccination.


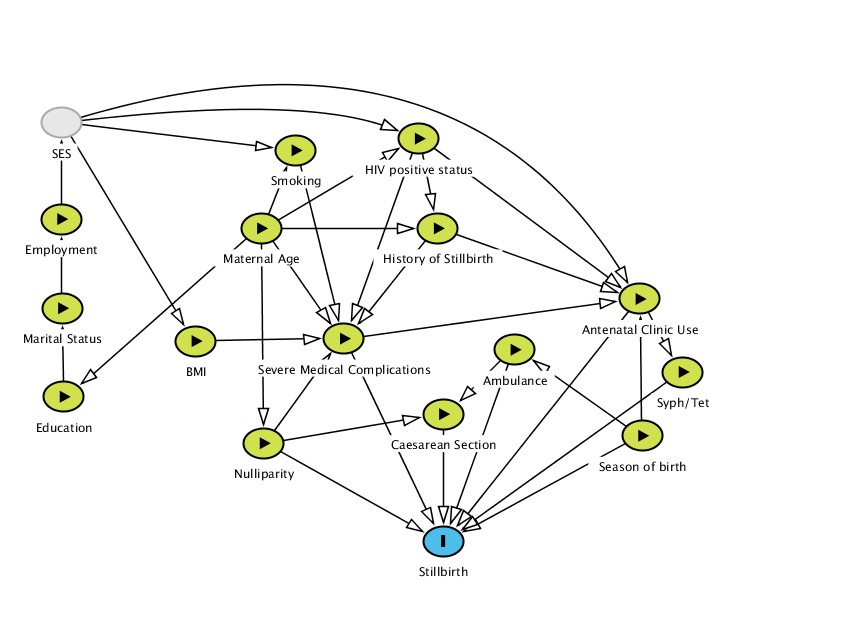

Supplement: Supplementary file 1 — Additional file 1: Supplementary Figure 1. Theoretical Directed Acyclic Graph to identify potential relationships between social, behavioural and medical factors and stillbirth. Factors in blue are outcome variables, those in green with an arrow are exposures of interest and those in grey are unmeasured factors. Diagram created in Dagitty Version Version 3.0.8 SES = Socioeconomic status, HIV – Human Immunodeficiency Virus, Syph/Tet – Syphilis serology and Tetanus vaccination. [file 12884_2021_4102_MOESM1_ESM.docx]
