# Supplementary material for: Identification of factors associated with stillbirth in Zimbabwe – a cross sectional study
Source: BMC Pregnancy Childbirth. 2021 Sep 29;21:662. doi: 10.1186/s12884-021-04102-y (PMC8482658; doi:10.1186/s12884-021-04102-y)
Supplement: Supplementary file 2 — Additional file 2: Supplementary Table 1. Singleton pregnancy model findings based on complete case data from postnatal ward sample (percentages exclude unknown cases). [file 12884_2021_4102_MOESM2_ESM.docx]

**Supplementary Table 1**. Singleton pregnancy model findings based on complete case data from postnatal ward sample (percentages exclude unknown cases)

|  | | **Livebirth** | **Stillbirth** | **Unadjusted CC OR (95% CI)** | **p-value** | **Adjusted CC OR (95% CI)** | **p-value** |
| --- | --- | --- | --- | --- | --- | --- | --- |
|  | | **N=1691** | **N=43** |  |  |  |  |
| Mother’s age in years | Mean (SD) | 26.8 (6.6) | 28.0 (7.1) | 1.03 (0.98-1.07) | 0.23 | 1.05 (0.94-1.18) | 0.38 |
|  | Unknown | 0 | 0 |  |  |  |  |
| Nulliparous | No | 1090 (64%) | 41 (95%) | 1 |  | 1 |  |
|  | Yes | 601 (36%) | 2 (5%) | 0.09 (0.01-0.29) | <0.001 | 3.77 (0.12-120.89) | 0.40 |
|  | Unknown | 0 | 0 |  |  |  |  |
| History of stillbirth | No | 1662 (98%) | 4 (9%) | 1 |  | 1 |  |
|  | Yes | 29 (2%) | 39 (91%) | 558.8 (208.7-1955.) | <0.001 | 4168.5 (456.2-130237) | <0.001 |
|  | Unknown | 0 | 0 |  |  |  |  |
| Number of antenatal care visits | > 4 | 533 (35%) | 6 (18%) | 1 |  | 1 |  |
|  | 1-4 | 861 (57%) | 18 (53%) | 1.86 (0.77-5.15) | 0.19 | 10.36 (2.00-77.50) | 0.01 |
|  | None | 110 (7%) | 10 (29%) | 8.08 (2.94-24.17) | <0.001 | 89.30 (10.24-1287.7) | <0.001 |
|  | Unknown | 187 | 9 |  |  |  |  |
| Season of birth | Mild | 1081 (64%) | 18 (42%) | 1 |  | 1 |  |
|  | Cold | 239 (14%) | 13 (30%) | 3.27 (1.55-6.72) | 0.001 | 16.64 (2.91-143.52) | 0.004 |
|  | Hot | 371 (22%) | 12 (28%) | 1.94 (0.90-4.03) | 0.08 | 1.65 (0.29-9.80) | 0.57 |
| C-section delivery | No | 1211 (72%) | 28 (65%) | 1 |  | 1 |  |
|  | Yes | 480 (28%) | 15 (35%) | 1.35 (0.70-2.52) | 0.35 | 0.30 (0.06-1.28) | 0.13 |
| Severe maternal complications | No | 1423 (86%) | 19 (54%) | 1 |  | 1 |  |
|  | Yes | 223 (14%) | 16 (46%) | 5.37 (2.69-10.60) | <0.001 | 9.52 (2.13-52.65) | 0.005 |
|  | Unknown | 45 | 8 |  |  |  |  |

CC: Complete case. OR: Odds Ratio. CI: Confidence Interval. SD: Standard Deviation.
